# A Crowd-Sourcing Indoor Localization Algorithm via Optical Camera on a Smartphone Assisted by Wi-Fi Fingerprint RSSI

**DOI:** 10.3390/s16030410

**Published:** 2016-03-19

**Authors:** Wei Chen, Weiping Wang, Qun Li, Qiang Chang, Hongtao Hou

**Affiliations:** College of Information Systems and Management, National University of Defense Technology, Changsha 410073, China; weichen@nudt.edu.cn (W.C.); wangwp@nudt.edu.cn (W.W.); qchang@telin.ugent.be (Q.C.); houhongtao@nudt.edu.cn (H.H.)

**Keywords:** fingerprint localization, optical camera, image processing, orientation sensor, crowd-sourcing, smartphone

## Abstract

Indoor positioning based on existing Wi-Fi fingerprints is becoming more and more common. Unfortunately, the Wi-Fi fingerprint is susceptible to multiple path interferences, signal attenuation, and environmental changes, which leads to low accuracy. Meanwhile, with the recent advances in charge-coupled device (CCD) technologies and the processing speed of smartphones, indoor positioning using the optical camera on a smartphone has become an attractive research topic; however, the major challenge is its high computational complexity; as a result, real-time positioning cannot be achieved. In this paper we introduce a crowd-sourcing indoor localization algorithm via an optical camera and orientation sensor on a smartphone to address these issues. First, we use Wi-Fi fingerprint based on the K Weighted Nearest Neighbor (KWNN) algorithm to make a coarse estimation. Second, we adopt a mean-weighted exponent algorithm to fuse optical image features and orientation sensor data as well as KWNN in the smartphone to refine the result. Furthermore, a crowd-sourcing approach is utilized to update and supplement the positioning database. We perform several experiments comparing our approach with other positioning algorithms on a common smartphone to evaluate the performance of the proposed sensor-calibrated algorithm, and the results demonstrate that the proposed algorithm could significantly improve accuracy, stability, and applicability of positioning.

## 1. Introduction

As the demands on location-based services (LBSs) increase, developing positioning systems (PSs) has generated great concern over the last decade; in particular, location information is an important basic feature in LBSs, and it is of great significance to get access to the users’ location information anytime and anywhere; in addition, users are more and more concerned about the performance of PSs, such as accuracy and stability. Outdoors, the Global Navigation Satellite System (GNSS), including GPS, GLONASS, Galileo, and BDS, which works well in most outdoor applications, is a major way to get users’ location information, and the accuracy is adequate for civilian use. Unfortunately, when users are in indoor environments, there exist some limitations, for example, lack of line of sight and multiple paths, both of which will cause satellite signal attenuation, so GNSS is not capable of providing services with sufficient positioning accuracy. At the same time, the widespread use and popularity of mobile devices have spurred extensive demands on indoor LBSs in recent years, which have greatly promoted the rapid development of entertainment, health, business, and other sectors, for example, users can easily get local information from the LBS when they are arriving at a place that is totally unfamiliar to them; likewise, when consumers want to go shopping in a big mall, when they arrive on-site equipment could send electronic coupons to them for advertising, two applications that without doubt, need to be based on precise indoor location information. To this end, indoor positioning systems have been widely developed, including Assisted GNSS (AGNSS) [1,2], Difference GNSS (DGNSS) [3,4], radio frequency identification (RFID) [5,6,7], WLANs [8,9,10], WSNs [11,12,13], optical sensors [14,15,16], and cooperative localization [17,18]. On the whole, no system exists that can adapt to the changing environment to meet different demands for localization due to their particular advantages and disadvantages.

With their great international popularity and embedded hardware sensors, smartphones have become the ideal personal and mobile navigator used to implement indoor positioning due to their low cost and self-contained properties. Meanwhile, Wi-Fi infrastructures are more and more generalized, indoor positioning systems based on Wi-Fi fingerprint and smartphones are widely applied, which includes the offline acquisition and online positioning stages. In the offline acquisition stage, the radio signal strength of reference points (RPs) is sampled to build a database, which is always time consuming and laborious; in the online positioning stage, the real-time received radio signal strength is used to locate with methods such as K-nearest neighbors (KNN) [19], K-weighted nearest neighbors (KWNN) [20], Bayesian [21,22], or artificial neural network (ANN) [23]. However, a Wi-Fi signal fluctuates because it is influenced by the complex indoor environment, which results in a low accuracy; besides, if the database of the Wi-Fi fingerprint cannot be updated in time, that is, the stored data cannot remain consistent with the actual signal, this will reduce the positioning accuracy.

Researchers have compared Wi-Fi fingerprint positioning with data from the built-in microelectronic mechanical systems (MEMSs) in smartphones, e.g., accelerometer, gyroscope, magnetometer, to improve Wi-Fi fingerprint positioning accuracy; these algorithms are well known as pedestrian dead reckoning (PDR) methods [20,24]. However, this technique has two drawbacks: on the one hand, it is only able to provide accurate data for a limited time because of the sensor errors arising from bias and noise, especially for the low-cost inertial sensors found in mobile phones, so the accumulating errors grow rapidly with the walking distance of pedestrians, on the other hand, we have tested a common smartphone and found that those sensors’ output is unstable and low accuracy, which will influence the positioning performance. Other researchers have also proposed positioning approaches combining Wi-Fi fingerprints and image matching [25,26,27,28]; these approaches can solve the problems mentioned above, however, a large-scale images database is required for those approaches, and the image processing and matching task is so time consuming that the required instantaneity is hard to guarantee in general.

Taking those advantages and disadvantages of stand-alone Wi-Fi fingerprints and stand-alone image matching positioning into account, this paper proposes a crowd-sourcing indoor localization algorithm via an optical camera on a smartphone to improve accuracy and instantaneity. First, we use the Wi-Fi signal fingerprint based on the KWNN algorithm to get the K nearest neighbors to make a coarse estimation, Second, we adopt a mean-weighted exponent algorithm with image features extracted by the Scale-Invariant Feature Transform (SIFT) operator and orientation sensor, whose output will constraint random images choice for a mobile node on a smartphone to refine the results based on a multithread mechanism. Compared with the stand-alone SIFT-based image matching positioning method, the proposed algorithm can reduce the search space of images and reduce the position estimation time. Compared with stand-alone Wi-Fi fingerprint positioning, the proposed algorithm can improve the accuracy, moreover, to prove effect of proposed algorithm further, we compare it with the visual positioning system based on the Speed-up Robust Feature (SURF) and KWNN algorithms used in literature [25]. Equally important, we utilize a crowd-sourcing method to update and supplement the database, which will retain consistency with the actual signal and obviously improve the stability of the positioning system. Experimental results show that the proposed algorithm could significantly overcome the disadvantages of stand-alone Wi-Fi fingerprint and stand-alone SIFT-based image matching positioning, and is more effective than SURF combined with KWNN positioning with respect to accuracy and stability. The proposed algorithm has been realized on an Android operating system smartphone, which has good research and application prospects.

The rest of the paper is organized as follows: Section 2 will review recent related works and summarize our contributions. In Section 3, we will present an overview of the proposed sensors-calibrated positioning system, including the offline acquisition and online positioning stages, then the proposed sensors-calibrated algorithm will be introduced in detail in Section 4, and the experimental results and evaluations will be shown in Section 5. Section 6 summarizes the whole paper, then provides our conclusions and an outlook of future work.

## 2. Related Works

Indoor positioning plays an important role in providing the location of people and objects. There are three principles used in indoor positioning: triangulation, scene analysis, and proximity. Triangulation locates the target point’s coordinates by using distances and angles between the target point and three other points with known coordinates, this principle includes Time of Arrival (TOA), Time Difference of Arrival (TDOA), Angle of Arrival (AOA), and Time of Flight (TOF). Scene analysis is a principle of positioning by analyzing the characteristics of the measured values, for example, received signal strength indicator (RSSI) is a popular scene analysis method to realize localization; available signals include Wi-Fi, radio frequency, geomagnetic, visible light, and so on. The proximity principle is mainly used in radio frequency-based positioning systems; in this technique, for a grid of antennas with fixed locations within a building, when a mobile node is detected, the closest antenna or the one receiving the strongest signal is selected to calculate the mobile node’s location [29], for example, cellular localization based on Cell-ID is one of the popular applications.

To meet the demanded high accuracy, some researchers have used hardware-based solutions to get a precise distance to locate objects accurately, which is a range-based method. For instance, Seybold [30] calculated the true distances between router and smartphone with a logarithmic-distance path-loss model. Zou [31] applied a weighted path-loss model to RFID signals to implement indoor positioning, on the basis of Zou, Chen [32] adopted a Kalman filter algorithm fusing received strength of Wi-Fi fingerprints, PDR, and landmarks to locate the position indoors. Fidan [33] used a geometric cooperative technique and path-loss model to estimate in real time the time-consuming coefficient of path-loss in ranging sensors, the simulation results indicated that the proposed approach can effectively adapt to uncertainty of the loss factor in signal propagation. Yang [34] and Kotaru [35] measured the phase–distance relationship of the radio signal to locate position, the experimental results show that those methods could reach an accuracy of decimeter level.

However, all of the mentioned solutions based on hardware above have some disadvantages and limitations from the aspect of practicability on a smartphone, for example, it is hard to synchronize the clocks between many common measuring devices, and the outputs of sensors on smartphones have low accuracy and stability in general, which will directly influence positioning performance. Finally, high-quality equipment will lead to increased cost, it is obviously not suitable for a smartphone.

In contrast, other researchers, including Google, Microsoft, Apple, *etc.* used software-based solutions to improve positioning performance, whose advantages are more flexibility, high accuracy, low cost, and short development cycle, and have developed some software-based positioning systems. Among those, Wi-Fi fingerprint is a common range-free way with great popularity, and various methods and theories about it have been researched to solve those issues and limitations mentioned in the Introduction. Schussel [36] and Li [37] used the actual signal and Gaussian distribution process to generate some fingerprints to fill the whole database. Through this approach, the positioning problem in different densities and environments was solved. To reduce computational complexity and positioning accuracy on the online stage, clustering theory has been used; Liu [38] proposed a dynamic constraint KNN algorithm by using indoor layout geometry information to cluster, however, the proposed algorithm required the extraction of features of the specific indoor area, so it is hard to promote its use. Saha [39] used the relative size of RSSI as a metric for clustering the fingerprint and tried to solve the equipment diversity issue in crowd- sourcing. The proposed algorithm can achieve a higher accuracy, but its processing involving clustering is also complicated. Lee [40] proposed a support vector machine-based clustering approach and used the margin between two canonical hyperplanes for classification; it reserved the fingerprint distribution property and obtained a superior decision boundary, but did not take instantaneity into consideration. In addition, the drawback of a time-consuming and laborious database prebuilding in the offline stage also needs to be overcome, the accuracy will decrease if the database is not being updated in time, thus, some researchers proposed a crowd-sourcing mechanism [41,42] to update and supplement the database.

To reduce development cost for the positioning system and increase its practicability, researchers have used smartphones’ built-in sensors to locate cooperatively. Keller [43] focused on the gyroscope and barometer in current smartphone to realize the indoor navigation with storey detection, because of their work, a position in three dimensions could be interpolated by the determination of a change of detected height with the knowledge of the relative motion distance or a prior known gradient of the stairs/ramp. Chen [32] utilized a Kalman filter to fuse Wi-Fi and sensors integrated in a phone as well as landmarks to finish locating; Galván-Tejada [44] adopted microphone, magnetometer, and light sensor to locate cooperatively; Shin [45] used Wi-Fi fingerprint and PDR to develop SmartSLAM, which locates pedestrians and constructs an indoor map at the same time. By using off-the-shelf smartphones and the methods mentioned above, they have realized indoor positioning, however, we have tested the output of sensors in common smartphones and found that the output values have low accuracy; even if we put the phone in a stationary state, the output fluctuated, therefore, we think that its low accuracy and stability will influence the performance of positioning systems.

Recent advances in charge-coupled device (CCD) sensor technologies have been applied to positioning and several visual positioning systems have been developed. Considering Wi-Fi fingerprint may have shadow areas where the Wi-Fi signal is weak, Song [46] proposed a new positioning technique by which captured images’ features and their coordinate information are stored to form a fingerprint, then they used the SURF algorithm along with Random Sample Consensus (RANSAC) to realize image similarity comparisons. Their method worked both indoors and outdoors, including the shadow areas where Wi-Fi fingerprint signals are weak. However, the time consumption was not discussed. Kawaji [47] utilized omnidirectional panoramic images and a PCA–SIFT algorithm to locate, with the proposed confidence factor locality sensitive hashing; this could improve the image matching performance and speed up image retrieval, however, the method required omnidirectional image acquisition and a higher performance processor, and could not be implemented on a mobile phone. Considering positioning accuracy, time consumption, automation and scalability, Levchev [28] proposed a simultaneous fingerprinting and mapping system, which used a particle filtering algorithm to fuse data sampled from sensors in smart mobile devices. Its Android application could achieve an average localization error of under 2 m, but its map and database generation process was complex and time-consuming; Further, a so-called Mobile Visual Indoor Positioning System (MoVIPS) using smartphone cameras was proposed by Werner [26] in 2011 and extended in 2014 [27]. MoVIPS used a dead reckoning approach and estimated orientation factors from smartphone compasses to reduce the amount of images, then quantized and clustered the SURF feature points, and used a transformation matrix to improve the distance estimation, a making it therefore faster, more precise and more efficient.

Visual positioning systems with image matching theories can improve the accuracy, unfortunately, it is generally necessary to construct an image database to be recognized, and the matching process is always time-consuming; in particular, these algorithms should be improved for use on a smartphone with ideal accuracy and instantaneity. Inspired by the researched literature, we propose our algorithm with the following contributions:

(1) In the preliminary positioning stage, we use a KWNN-based Wi-Fi fingerprint positioning algorithm to make a coarse estimation, then select K images corresponding to K-nearest neighbors as retrievable images to reduce the time spent in retrieving images.
(2) After Wi-Fi fingerprint positioning, we introduce an orientation matching factor calculated using a three-dimensional orientation sensor on a smartphone to constrain random images choice for a coarse estimated location, then, we use the SIFT operator to extract image features to calculate the image matching factor on a multithread mechanism.
(3) We propose a mean-weighted exponent algorithm to improve accuracy by fusing the KWNN algorithm, orientation matching factor, and image matching factor.
(4) We use crowd-sourcing to update the database; users can upload their positioning results to a server, which can aids others’ positioning the next time if the database has not recorded some RPs information in the offline stage.
(5) Our algorithm has been realized in an Android operating system smartphone, and is easy to implement and apply. There is no need to use additional hardware assistance and the costs are greatly reduced.

## 3. Overview of the Proposed Sensors-Calibrated Positioning System

We propose a crowd-sourcing indoor localization algorithm via an optical camera and orientation sensor on a smartphone, with the purpose of simplifying indoor localization with high accuracy through the use of a Wi-Fi module and camera as well as the orientation sensor in the smartphone, and it does not need to be assisted by other hardware. The proposed system includes two stages: offline acquisition and online positioning stages. In the offline stage, the smartphone will scan available access points (APs) at different RPs, then record the RP’s coordinates, AP’s MAC address, and its RSSI values, while recording the front image of RPs and output values of the three-dimensional orientation sensor. In this paper, we use a relational database whose format is shown in Table 1 to store those data, and those images will be preprocessed in a server using the SIFT operator and extracting the features vector. 

**Figure 1:**
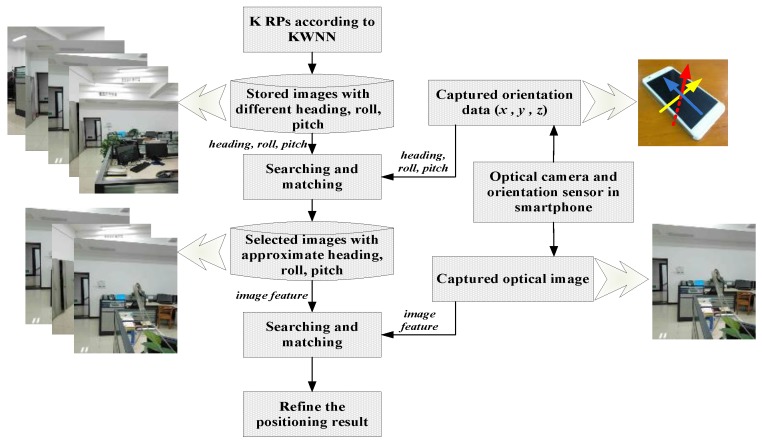
Framework of positioning calibration using built-in sensors in a smartphone.

In the online positioning stage, users open the Wi-Fi and scan accessible APs, record the MAC address and RSSI values in the cache of the phone, then users can choose to use Wi-Fi fingerprint positioning based on the KWNN algorithm to perform rough localization. In order to achieve higher accuracy, we use the built-in sensor to calibrate the positioning result of Wi-Fi fingerprint. The framework of this approach is shown in Figure 1. Users can open the camera to take a picture, and the three-dimensional orientation values will be recorded in the cache simultaneously. The features vector are extracted from the captured images and recorded temporarily in the phone cache. The orientation values will be used to calculate the orientation factor, which can restrain random image choices at a position. The temporary features vector and stored features vector corresponding to the K nearest neighbors are used to calculate the image matching factor, then the mean-weighted exponent algorithm will fuse KWNN, orientation matching, and image matching results to improve the accuracy further. At the same time, the users can upload the results and those temporarily stored data including MAC address, RSSI, image feature, and orientation values into a server to update the database and aid better location estimate of others the next time.

Table 1 shows the recording format of RPs’ coordinates, Wi-Fi fingerprint, captured image sequence number, and output values of the orientation sensor. The coordinates are specified by the user when building the database in the offline stage, but when using crowd-sourcing to update, they are calculated by our proposed algorithm. The AP number means the total number of available APs when users scan Wi-Fi, it is worth noting that the AP number of different RPs are not all equal because of the changing environment at different places, thus, m_1_, m_2_, ..., m_n_ in Table 1 are not all equal. AP_1_–AP_m_ record the received APs’ MAC and RSSI; when making a coarse estimation, the KWNN algorithm only calculates the distance of RSSI with the same MAC address. Image sequence number is recorded for 1 to n, and the image features are stored in another data table named as the corresponding sequence number of images. Because of the random direction when users take a picture, namely, it is likely that users take pictures at the same place but the direction of the image is different, so in this paper we use orientation factor to restrain this case of randomness.

## 4. Crowd-Sourcing Localization Assisted by Wi-Fi Fingerprints

### 4.1. Wi-Fi Fingerprint Positioning Based on KWNN

K-weighted nearest neighbors is a version of the K-nearest neighbors (KNN) algorithm improved by introducing a weighted distance factor. If the RPs’ distribution is sparse or non-uniform, after calculating the distances and selecting K nearest neighbors, the classification result of KNN is worse than that of KWNN. Torres-Sospedra’s [8] extensive experimental results showed that Euclidean distance is not the optimal choice to describe the similarity between fingerprints. On the contrary, the Sorensen distance function can achieve a better accuracy than Euclidean distance. Therefore, in this paper, we choose the Sorensen function for the KWNN algorithm. The definition of the Sorensen distance function is:

(1) $$L_{\left(d,i\right)}^{}=\frac{\sum_{i=1}^{n}\left|RSSI_{MN}^{\left(i,n\right)}-RSSI_{RP}^{\left(i,n\right)}\right|}{\sum_{i=1}^{n}\left|RSSI_{MN}^{\left(i,n\right)}+RSSI_{RP}^{\left(i,n\right)}\right|}$$

where *i* is a sequence of RPs, n denotes the number of APs with the same MAC between mobile node and RPs, MN denotes mobile node, RP denotes a reference point, then, the K-nearest distance will be selected as the nearest neighbors. Finally, the distances corresponding to the K neighbors are used to calculate the normalized weights using [20]:

(2) $$\omega_{i}=\frac{1/L_{\left(d,i\right)}^{}}{\sum_{i=1}^{K}(1/L_{\left(d,i\right)}^{})}$$

According to the normalized weight and RPs’ coordinates, a coarse estimation using the KWNN-based Wi-Fi fingerprint can use the following formula [20]:

(3) $$P_{i}{\left(x,y\right)}_{KWNN}=\{\sum_{i=1}^{K}x_{i}\cdot \omega_{i},\sum_{i=1}^{K}y_{i}\cdot \omega_{i}\}$$

**Figure 2:**
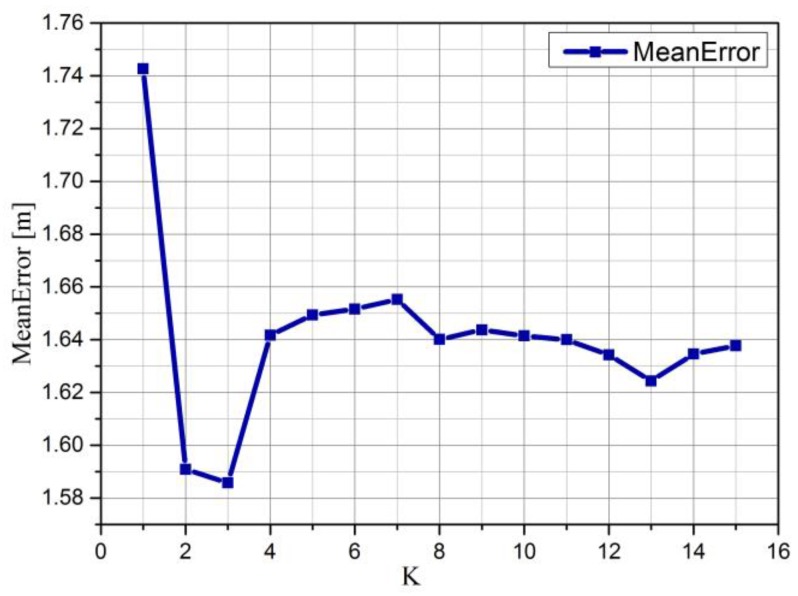
The K value’s impact on positioning.

In KWNN, it is well known that K is a critical parameter that will directly influence the positioning accuracy, so we have explored that and the results are shown in Figure 2. Each K was simulated 1000 times to calculate the mean error of positioning; we can see from Figure 2 that K = 3 is the optimal choice whose mean error is about 1.59 m, so we set K as 3 in this paper, which will determine the number of retrievable images.

### 4.2. Precise Localization Calibrated with Built-in Sensors on a Smartphone

At the image matching positioning stage, the images and three-dimensional orientation sensor data captured via the user’s smartphone will be matched with the stored data according to the K nearest neighbors. This process can be described like this: first, we calculate the angle difference between real-time obtained orientation values and stored values, and the results are expressed as orientation matching factors, afterwards, the captured image will be processed to calculate the image matching factor. It is worth noting that the orientation matching factors are three-dimensional vectors, but the image matching factors are 1 × K vectors, where K is the parameter in the KWNN algorithm. Finally, we will utilize the mean-weighted exponent algorithm to correct further the estimated results of the Wi-Fi fingerprint. More pertinent details will be introduced in the following sections.

#### 4.2.1. Three-Dimensional Orientation Matching

**Figure 3:**
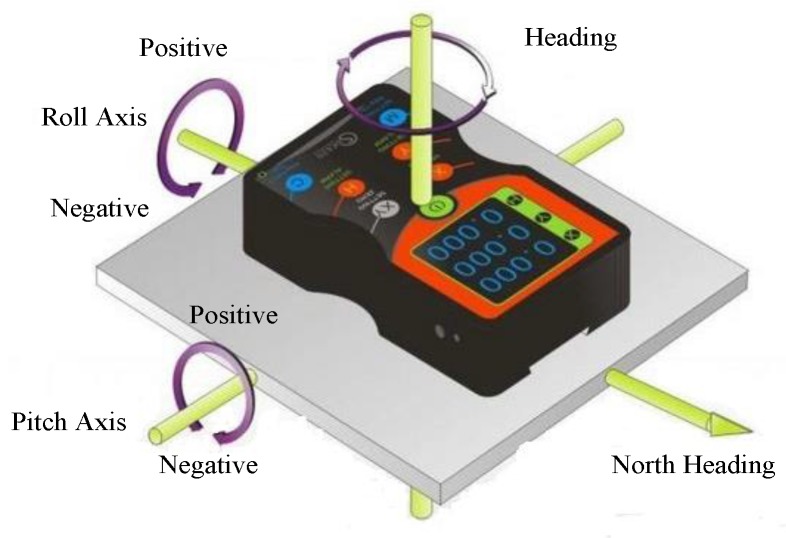
3D model of an orientation sensor.

The orientation sensor is used to detect motion pose such as azimuth, pitch and roll. We show the 3D model of orientation sensor in Figure 3, it is software-based and derives its three-dimensional floating data from the accelerometer sensor and the geomagnetic field sensor using rotation matrix. Since this whole process has been packaged into the Android API, we can use it directly.

**Figure 4:**
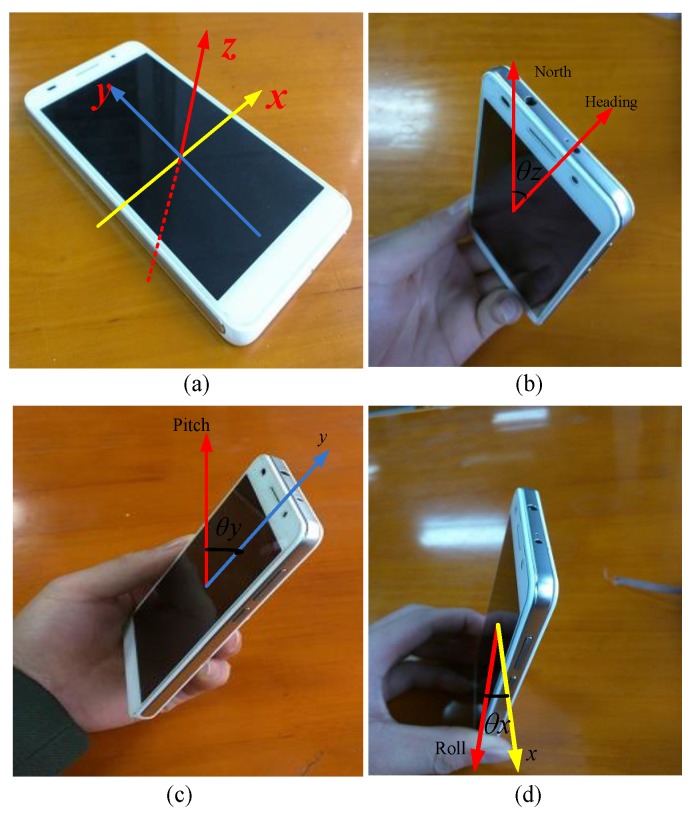
(**a**) The coordinate system in a smartphone; (**b**) heading indication of a smartphone; (**c**) pitch indication of a smartphone; (**d**) roll indication of a smartphone.

We use the orientation sensor in a smartphone to improve the image matching performance, so we should know its coordinate system shown in Figure 4a, where the z-axis represents azimuth or heading. When the phone rotates around the z-axis, the first element in the three-dimensional floating data, whose range is 0–360°, indicates the angle between the geomagnetic field and the phone’s y-axis, so if the value is equal to 0°, 90°, 180°, or 270°, it means the heading is north, east, south, or west, respectively, and we show this case in Figure 4b. The x-axis represents pitch. When the phone rotates around the x-axis, the second element in the three-dimensional floating data, whose range is −180° to +180° indicates the angle between the geomagnetic field and the phone’s z-axis, specifically, when the top of the phone is up, the angle changes from 0° to −180°, when the bottom of the phone is up, the value changes from 0° to 180°. This case is shown in Figure 4c. The x-axis represents roll. When the phone rotates around the y-axis, the second element in the three-dimensional float data, whose range is −90° to +90° indicates the angle between the geomagnetic field and the phone’s x-axis, specifically, when the left of the phone is up, the angle changes from 0° to −90°, when the right of phone is up, the value changes from 0° to +90°. This case is shown in Figure 4d.

In our algorithm, when the user takes a picture, the output values of the orientation sensor and the image will be recorded into the database simultaneously. The angle difference between real-time obtained orientation values and stored values can improve the stability of image matching performance, namely, the values are used to constrain random image choices for a coarse estimated location. A smaller angle difference indicates that the direction of the user and the pose of the smartphone are closer to the corresponding RPs. In this paper, we define the orientation matching factor to measure the closeness as follows: assume that the real-time obtained three-dimensional orientation values are represented as { θx,θy,θz }, the stored values acquired from the K nearest nodes are denoted as { θxi,θy,iθzi }, then, the angle difference can be calculated using the following formula:

(4) $$\Delta \theta_{i}=\sqrt{{\left(\theta x-\theta x_{i}\right)}^{2}+{\left(\theta y-\theta y_{i}\right)}^{2}+{\left(\theta z-\theta z_{i}\right)}^{2}}$$

Finally, the orientation matching factor is the normalized weights of the reciprocal value of the angle differences:

(5) $$\zeta_{i}=\frac{1/\Delta \theta_{i}}{\sum_{i=1}^{K}\left(1/\Delta \theta_{i}\right)}$$

where *i* = 1, 2, …, K indicates the sequence of RPs in Equations (4) and (5).

#### 4.2.2. Image Feature Matching by an Optical Camera

**Figure 5:**
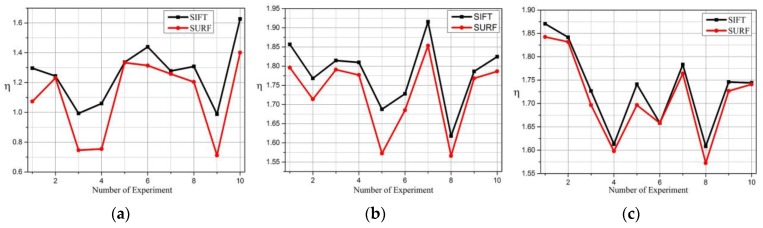
(**a**) the impact of scale changes on image matching factor; (**b**) the impact of rotation on image matching factor; (**c**) the impact of blur on image matching factor.

Image feature matching has been researched for indoor localization for years, and image features can be extracted with many algorithms, for example, SIFT, PCA-SIFT, SURF. Niu [25] and Luo [48] compared SIFT and SURF from the perspective of time, scale, rotation, blur, illumination and affine. We have tested this in our experiments and it proves that SURF is more than three times faster than SIFT, however, it is not optimal to handle scale changes, rotation transformation and blur; a performance comparison of those three changes is shown in Figure 5. 

Considering that there exists scale, rotation and blur changes when users take photos in many cases, thus, we choose the SIFT algorithm proposed by Lowe [49] to extract image features. Extracted features can be expressed as N × 128-dimensional feature vectors and stored in a database, where N denotes the total number of obtained feature points. The image captured on a smartphone by users is processed by the same algorithm, then we calculate one by one the Sorensen distances between the stored feature vectors and the real-time captured ones, we calculate the ratio by comparing the distance of the closest neighbor to that of the second-closest neighbor as the metric for similarity of images. Lowe demonstrated that a metric threshold of “feature distance” between 0.4 and 0.6 is optimal when the two matching images change with scaling change and rotation transformation [49], further, we have found in our many experiments that when the threshold is equal to 0.55, the positioning mean error is smallest, the result is shown in Section 5.2.1. The process of image matching is as follows: it is based on the condition that we have obtained K images after coarse positioning with the KWNN-based Wi-Fi fingerprint, where K is the number of nearest neighbors. As mentioned above, the amount of database images in the candidate set has been reduced and the randomness of images choice have been restricted with the orientation sensor. To decrease the computational time, K images will be processed in a multithread mechanism.

Those K images corresponding to K nearest neighbors in the database can be expressed as $I_{\left(DB,i\right)}$ (*i* = 1, 2, ..., K), and feature vectors are indicated as $F_{\left(DB,i\right)}$ (*i* = 1, 2, ..., K), the image captured by users is represented as Is and its corresponding feature vectors can be donated as Fs, then we use the following formula to calculate the common feature vectors Fc between the captured image and stored images:

(6) $$Fc=(F_{\left(DB,i\right)}\cap Fs)\in [0,min(F_{\left(DB,i\right)},Fs)]$$

where *i* = 1, 2, ..., K. After that, two ratios between those images can be calculated, there are ( $\left(F_{\left(DB\;,\;i\right)}\cap \;F_{s}\right)/F_{\left(DB\;,\;i\right)}$ ) and ( $\left(F_{\left(DB\;,\;i\right)}\cap \;F_{s}\right)/F_{S}$ ). Accordingly, we use the two ratios to calculate the image matching factor. This factor can be defined as:

(7) $$\eta_{i}\Rightarrow arg\;max(\frac{F_{\left(DB,i\right)}\cap Fs}{F_{\left(DB,i\right)}}+\frac{F_{\left(DB,i\right)}\cap Fs}{Fs})$$

where *i* = 1, 2, 3 denotes the index of the selected images. The image in the database that should be picked up if it maximizes the image matching factor, as we can see, if the two images don’t contain common feature points, the η is 0, if the two images match totally, the η is 2, so the range of η values is between 0.0 ≤ η ≤ 2.0, and a larger η indicates a greater similarity between two images.

#### 4.2.3. Precise Localization Using Mean-Weighted Exponent Algorithm

In this section we will introduce our fusion method, which we name the mean-weighted exponent algorithm, to calibrate the estimated location through the KWNN algorithm. The reason why we chose an exponent function is due to its nonlinearity, namely, an exponent function has a more significant change rate compared with a linear function.

According to the selected K RPs, we have calculated the distance factor ω by KWNN, and the orientation matching factor ξ as well as image matching factor η through the proposed algorithm. With those critical parameters, we fuse them by calculating the sum, which will be used as the power of the exponent function. Similar to the previous principle, the normalized correction factor after fusion is represented as:

(8) $$\xi_{i}^{\prime }=\frac{e^{\left(\omega_{i}+\lambda \eta_{i}+\gamma \zeta_{i}\right)}}{\sum_{i=1}^{K}e^{\left(\omega_{i}+\lambda \eta_{i}+\gamma \zeta_{i}\right)}}$$

where K is the number of nearest neighbors, *i* = 1, 2, …, K, λ and γ denote adjustment parameters. In this paper, we set both λ and γ to 1. Finally, the location of the user improved by the camera and orientation sensor on the smartphone is estimated by utilizing the following formula, similar to Equation (3):

(9) $$P_{i}{\left(x,y\right)}_{Fianl}=\{\sum_{i=1}^{K}x_{i}\cdot \xi_{i}^{\prime },\sum_{i=1}^{K}y_{i}\cdot \xi_{i}^{\prime }\}$$

Equally important, after calculating the orientation matching and image matching factors, it should be noted that the orientation matching factors are three-dimensional vectors, but the image matching factors are 1 × K vectors, where K is the parameter in the KWNN algorithm. Then, we first set thresholds to judge the maximum value of those factors, if the maximum values are both greater than the two thresholds, this indicates that the location of the reference point corresponding to that maximum value can be used as the final location of the user. In this paper, they are equal to 1.8 and 0.8.

### 4.3. Updating the Database by Crowd-Sourcing

One of the disadvantages of existing Wi-Fi fingerprint positioning is the very high costs in time and manpower during the offline acquisition and calibration phases. To overcome these limitations, our proposed algorithm supports users in uploadiang their scanned Wi-Fi data and captured images into a server to update the database, that is, crowd-sourcing, Estelles-Arolas [50] overviewed this and defined the idea as “a new online distributed production model in which people collaborate and may be rewarded to complete tasks” [51], in our design, we always assume the users are willing to upload the right results into the database. Apart from the fact that it can greatly reduce the complexity of building the database, we can find other advantages from our experiment in that the accuracy of both the Wi-Fi fingerprint and proposed sensors-calibrated system positioning results are higher and more stable as the amount of data in the database increases, as the results in Section 5.2.3 show, so users contributing to crowd-sourcing can get a more precise and stable results. Moreover, it can aid others to locate the next time if the database has not recorded some RPs information in the offline stage, in this way, it also decreases the manpower cost.

## 5. Experimental Results and Evaluation

### 5.1. Experimental Setup

**Figure 6:**
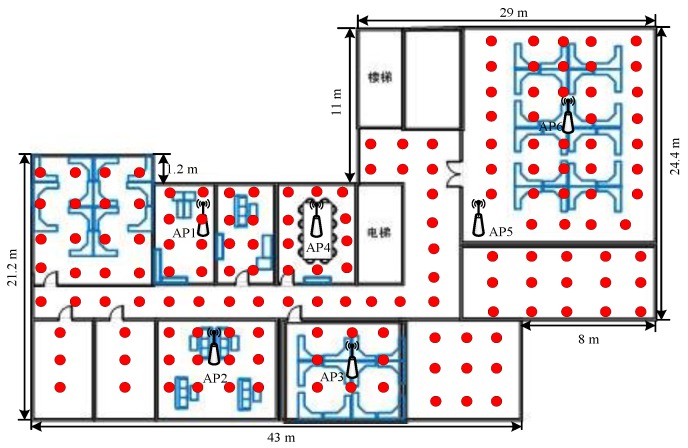
Layout and RPs.

**Figure 7:**
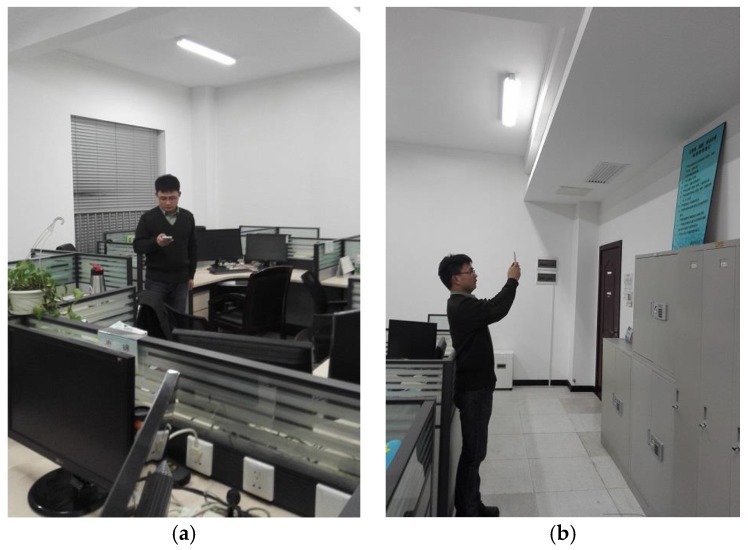
Scanning WiFi fingerprint (**a**) and capturing images (**b**).

To validate our proposed algorithm, we have developed an application on an Android smartphone. Our experiment was performed on the fourth floor at the College of Information System and Management of NUDT, with an area of around 1250 m^2^ containing six fixed APs, the layout of floor and RPs installation are shown in Figure 6. During the experiment, we scan the Wi-Fi signal RSSI and record the captured front images and three-dimensional orientation values to build the database shown in Figure 7. We did our best to try to capture fixed icons, such as signs, house numbers, *etc.* We collected 150 RPs’ information in the offline stage in the first round. In our experiment, our experimental hardware included: a TP-link router, ThinkPad E420 with Win 7 64-bit operating system, Intel(R) i3-2310M CPU 2.10 GHz 4 cores and 4 G memory. The smartphone is a Huawei Honor 6 (H60-L01) mobile 4G, 16 G ROM whose sensors parameters are listed in Table 2 and Table 3, moreover, we used a Kingston 8 GB micro SD memory card to store data in the phone. We developed our software using Android Developer Tools v 21.1.0.

**Figure 8:**
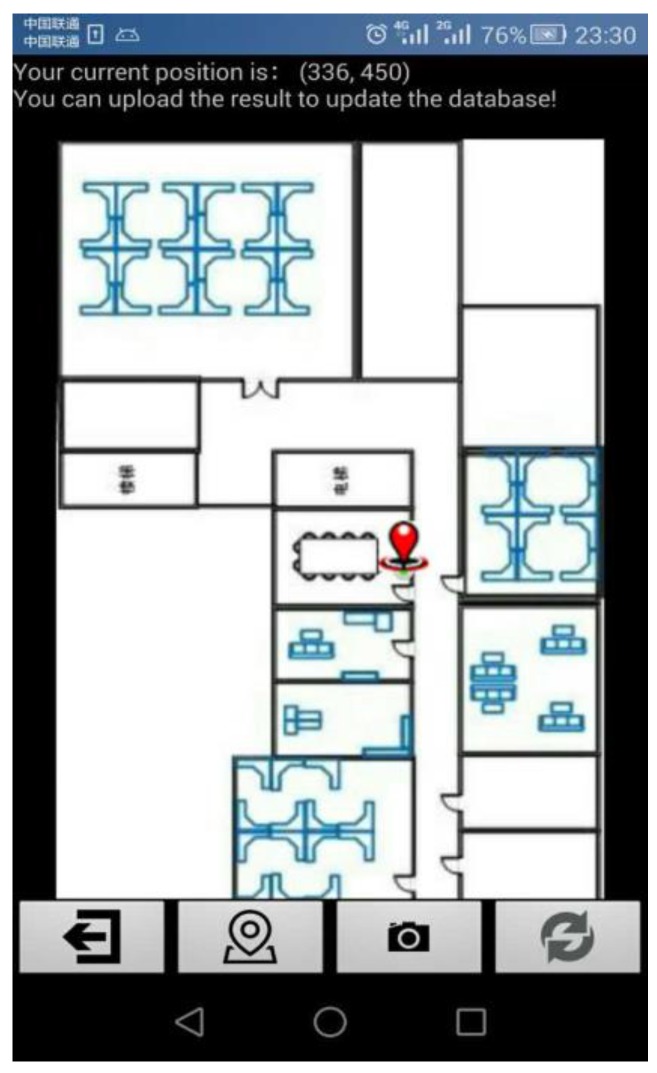
Display interface and positioning result.

The simple interface of the app and positioning result are displayed in Figure 8. The red point represents the current location, and a prompt message is at the top.

Four buttons are shown in the bottom, from left to right, their function is: exit, Wi-Fi positioning, image matching positioning, and upload data to the server. Clicking the Wi-Fi positioning button uses the KWNN algorithm to estimate the position for users, After the KWNN algorithm implements the positioning, clicking the image matching positioning opens the camera automatically and further corrects the roughly estimated result using our proposed algorithm. Clicking the upload data button uploads the data and the refined localization in the phone cache into the database. Clicking exit exits our positioning application software. Initially, the user can point to a location on the map and upload the current data into the server to build a database.

### 5.2. Experimental Results and Performance Evaluation

#### 5.2.1. Image Matching Evaluation

**Figure 9:**
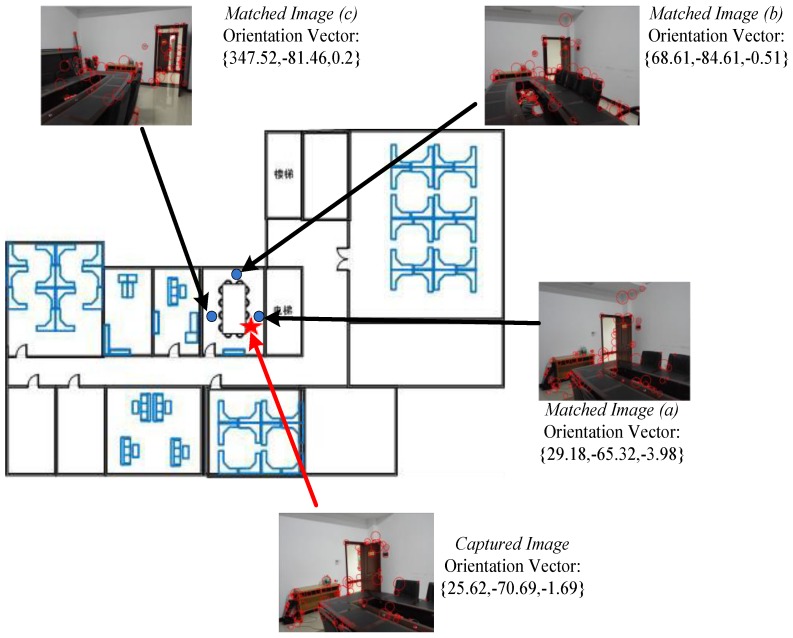
Comparison of image and orientation matching.

An experiment is conducted in the meeting room shown in Figure 9, in which we display the captured image and matched images (a), (b), and (c) through KWNN, as well as corresponding orientation vectors. The red pentagram indicates the current position and its captured image, scanned APs’ RSSI value are {−63,−44,−85,−94,−89,−86}, the other three RSSI values stored in the database are {−67,−46,−90,−80,−81,−86}, {−73,−38,−85,−80,−83,−80}, and {−75,−45,−88,−82,−88,−81}. The captured orientation sensor data is {25.62,−70.69,−1.69}, the other three stored in the database are {29.18,−65.32,−3.98}, {68.61,−84.61,−0.51} and {347.52,−81.46,0.2}. In our experiment, we compressed all images with the size of 1034 × 731, the image captured by the user at the online stage is processed with the SIFT operator and the number of extracted features *Fs* = 714. We list the other three images’ features and common features in Table 4, meanwhile, we calculate those factors using the methodology described in Section 4.

**Figure 10:**
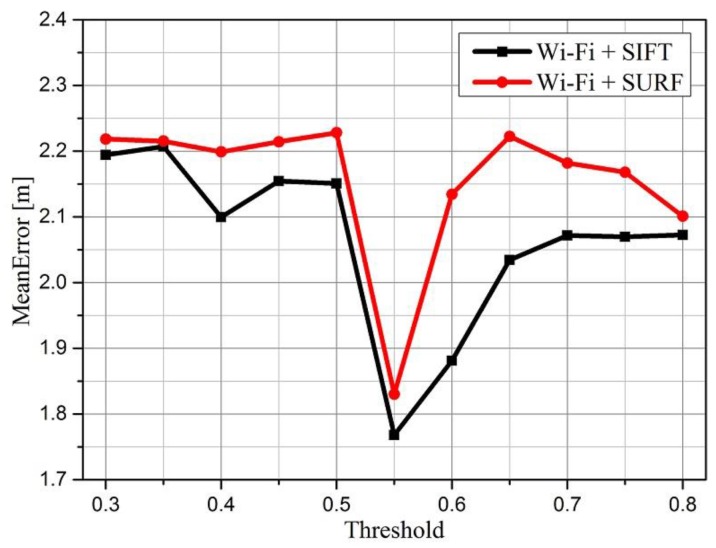
Different thresholds’ impact on positioning accuracy.

It is well known that Lowe demonstrated that a metric threshold for Euclidean distance between 0.4 and 0.6 is optimal [49,52]. In this case the probability density functions for correct matches are obviously higher than for incorrect matches. In our experiment, we have explored the metric threshold’s impact on the positioning accuracy of SURF and SIFT algorithm, it worth noting that “Wi-Fi+SURF” means the method using Wi-Fi fingerprint to make a coarse estimation and SURF-based image matching algorithm to refine the positioning results, which was used in [25], “Wi-Fi+SURF” is the proposed algorithm in our paper. The results are shown in Figure 10, where we selected 150 RPs in the database, and located each group 200 times with different thresholds. As we can see clearly, the mean error is lowest when the threshold is set to 0.55 both of the two algorithm, however, the mean error increases significantly when the threshold is greater than 0.75, the accuracy becomes worse, consequently, we set the threshold to 0.55 when using feature vector to match images and realize positioning in the online stage.

#### 5.2.2. Positioning Evaluation

**Figure 11:**
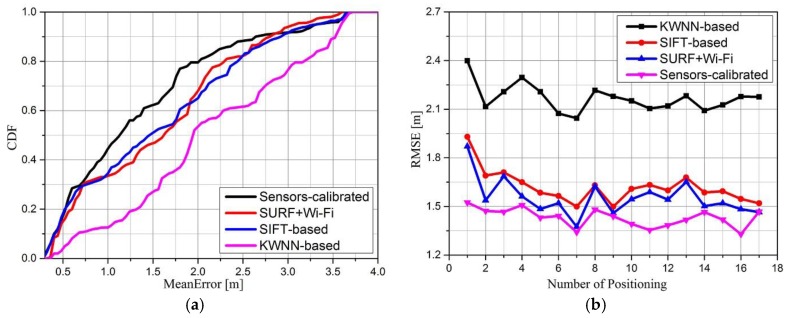
Performance comparison of our sensors-calibrated algorithm with other approaches.

To validate the accuracy and correctness of the proposed algorithm with calibrated sensors, we have performed an experiment with 150 RPs in the database. The Cumulative Distribution Function (CDF) and root mean square error (RMSE) of the stand-alone KWNN-based Wi-Fi fingerprint algorithm, stand-alone SIFT-based positioning algorithm, SURF combined with Wi-Fi positioning algorithm and proposed sensors-calibrated algorithm are shown in Figure 11. In Figure 11a, we count the errors of 200 positioning events and we can see that positioning accuracy could be greatly improved by the sensors-calibrated algorithm compared with other approaches, for example, the error probability distribution of the KWNN-based algorithm under 1 m is about 12%, under 2 m is about 53.5%, these results demonstrate that the accuracy of the KWNN-based algorithm is not high enough. However, with correction of optical image and orientation sensor matching, this probability distribution increases to 43%, the probability under 2 m is about 80%. The stand-alone SIFT-based algorithm also has a high error probability distribution, and these results demonstrate that the accuracy of the KWNN-based algorithm is improved by visual positioning algorithm obviously (*i.e.*, the sensors-calibrated algorithm). Significantly, the correctness of the KWNN directly impacts the accuracy of the sensors-calibrated algorithm. The RMSEs of different algorithms are shown in Figure 11b, where the RMSE of the sensors-calibrated algorithm is between 1.2 m and 1.5 m, less than the RMSE of the other three positioning algorithms. The stand-alone SIFT-based and “SURF+Wi-Fi” method have close RMSE results, while the stand-alone KWNN-based algorithm is worse, ranging from 2.1 to 2.4. We can conclude that our proposed sensors-calibrated algorithm has better performance than the others and the estimated results are closer to the true location after calibration.

#### 5.2.3. Performance of Crowd-Sourcing for Positioning

**Figure 12:**
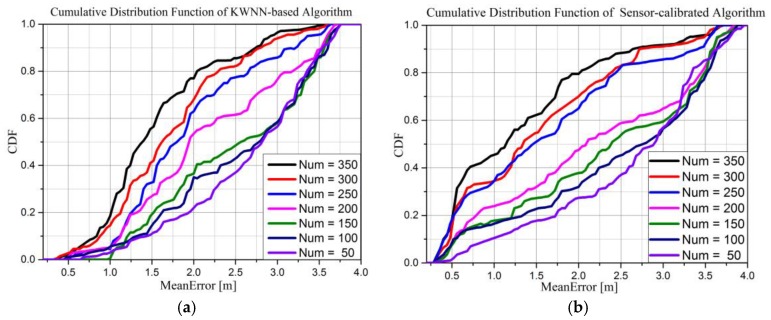
Performance comparison of crowd-sourcing.

We use the error CDF and accuracy as the standard to evaluate the performance of crowd-sourcing. In our small-scale experiment, five people participated in uploading data on the fourth floor at the College of Information System and Management of NUDT. We assume users participate actively, uploading positioning results freely and honestly, that is, there is no unreliable data in the experiment. In this section, we select compare the performance of the stand-alone KWNN algorithm and our proposed algorithm. The number of items in the database is set to 50, 100, 150, 200, 250, 300, and 350 to simulate the increase in the database size through crowd-sourcing. The results are shown in Figure 12.

Figure 12a,b show the error CDF of the KWNN-based and the proposed sensors-calibrated algorithm, respectively. From Figure 12 we can conclude that:

(1) As the number of RPs in the database increases, the accuracy of the KWNN-based and our sensors-calibrated algorithm are both improved.
(2) The sensors-calibrated algorithm has a better performance. For example, when the number of RPs is equal to 350, the mean error under 1 m of KWNN-based algorithm is about 20%, but, the mean error under 1 m of the proposed algorithm is about 46%, an obvious improvement.
(3) For the same database, if the estimated results of the KWNN-based algorithm are more precise and more stable, which will choose better nearest neighbors, so finally, the accuracy of our sensors-calibrated algorithm will be better.

**Figure 13:**
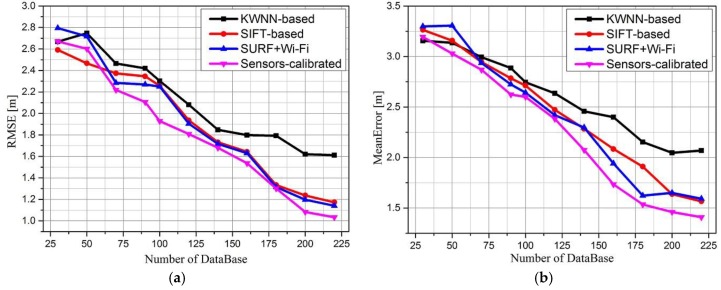
RMSE and mean error comparison of crowd-sourcing with different algorithms.

The RMSE and mean error of the stand-alone KWNN-based Wi-Fi fingerprint algorithm, stand-alone SIFT-based positioning algorithm, SURF combined with Wi-Fi positioning algorithm and proposed sensors-calibrated algorithm are shown in Figure 13, where the mean error indicates the closeness between the average value of a number of measurements and an accepted reference value, namely, trueness. 

The RMSE indicates a measure of the system’s combined trueness and precision, namely, accuracy. Each group was performed 200 times. Figure 13a compares the RMSE of these four algorithms, Figure 13b compares the mean errors of these four algorithms. We can conclude the following from the shown results: as the number of RPs increases, the RMSE of the KWNN-based algorithm decreases from 2.8 m to 1.6 m, reducing by 42.9%. The sensors-calibrated algorithm decreases from 2.7 m to 1.02 m, reducing by 62%, stand-alone SIFT image matching algorithm and SURF combinated with Wi-Fi algorithm decrease 54.2% and 57.9%, respectively.

The RMSE of the sensors-calibrated algorithm is less than that of the other three algorithms, which means the position from the sensors-calibrated algorithm is closer to the true location, while the stand-alone SIFT-based algorithm and SURF combined with Wi-Fi algorithm results are approximate.

From Figure 13b, we can see that the mean error of visual positioning algorithm is less than that of KWNN-based Wi-Fi fingerprint positioning, and with the coarse estimation by stand-alone KWNN-based algorithm, the proposed sensors-calibrated algorithm is less than those of the other three algorithms as the number of RPs increases, and the rate of increase of the proposed algorithm is larger than that of the others, which indicates the sensors-calibrated algorithm has a better performance.

**Figure 14:**
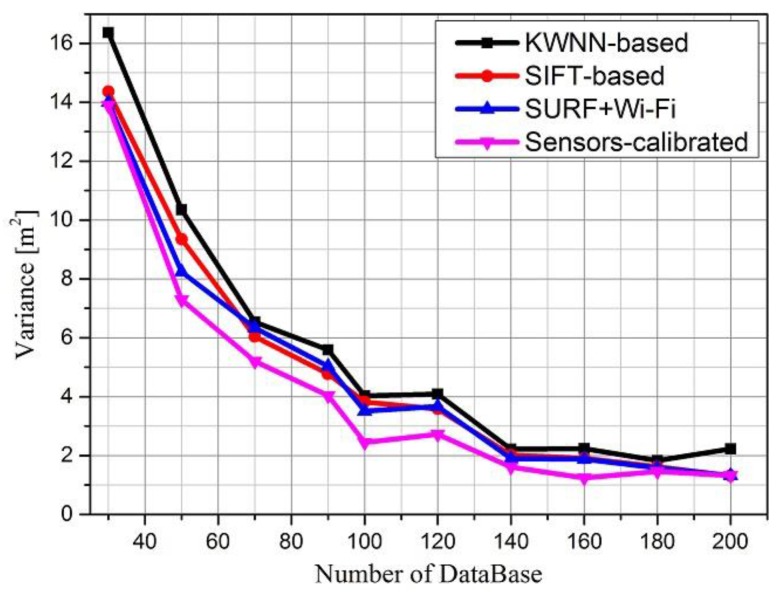
Positioning variance comparison.

We can use data variance to evaluate the stability of positioning. Positioning variance comparisons of the different algorithms are shown in Figure 14. As we can see, when the number of RPs increase, the variance of the four algorithms decreases, however, the positioning variance of the proposed sensors-calibrated algorithm is less than the variance of other algorithms, stand-alone SIFT-based algorithm and SURF combined with Wi-Fi algorithm are approximate, therefore, the results demonstrate that the proposed algorithm is more stable than KWNN.

#### 5.2.4. Performance Comparison of Time Consume for Positioning

Time consumption is an important aspect for positioning systems, and it’s well known that the SURF algorithm is faster than the SIFT algorithm. In our proposed sensors-calibrated algorithm, we use a multithread mechanism to extract features of the K stored images corresponding to the K nearest neighbors in parallel by using the SIFT operator, so the proposed algorithm’s time consumption for estimating positions of is not worse than SURF, but the time consumption of a stand-alone SIFT-based algorithm is almost three times that of our proposed sensors-calibrated algorithm, and the position estimation time increases greatly as the image database increases in size, thus, we show the results of stand-alone KWNN-based, SURF combined with Wi-Fi algorithm and the proposed algorithm (*i.e.*, SURF+Wi-Fi) in Table 5.

As we can see, the time consumption of the KWNN-based algorithm is the least; the average is 23.79 ms. As we put more emphasis on the time consumption of image matching positioning after coarse estimation by the KWNN-based algorithm, with the help of the multithread mechanism, the average time consumed by our proposed algorithm is 1716.20 ms. At the same time, the time consumption of the image matching approach based on SURF is 1808.03 ms, so the results demonstrate that high computational cost of image matching positioning have been reduced greatly by our sensors-calibrated algorithm.

## 6. Conclusions

Aiming at solving the imprecise and time-consuming outcomes in Wi-Fi fingerprint and image matching positioning, respectively, in this paper we propose a crowd-sourcing indoor localization algorithm via the optical camera and orientation sensor in a smartphone. Real-case experimental results of different methods demonstrate that the sensors-calibrated algorithm has a better accuracy and stability performance, compared with three other algorithms (*i.e.*, stand-alone KWNN-based positioning, stand-alone SIFT image matching positioning and SURF combined with Wi-Fi positioning). In summary, the proposed sensors-calibrated crowd-sourcing indoor localization algorithm via the optical camera and orientation sensor on a smartphone has three innovative aspects:

(1) We explore the impact of SIFT and SURF thresholds on positioning when we choose the SIFT operator to extract image features. We conclude from our experiments that the positioning error is the least when the threshold is set as 0.55 in both the SIFT and SURF algorithms.
(2) We propose a mean-weighted exponent algorithm to fuse the output of the built-in sensors in a smartphone. Small scale real-case scenarios results demonstrate that the proposed algorithm has a better performance because of its nonlinearity to calibrate the positioning accuracy.
(3) We use crowd-sourcing to update and supplement the database. We also explored its influence on positioning. The experimental results show that the accuracy and stability of both algorithms have been improved, and the sensors-calibrated algorithm is obviously better than other three algorithms in this paper.

Compared with the other positioning systems, the proposed system has the following advantages:

(1) *Higher performance*. We apply the mean-weighted exponent algorithm to calculate the correction factors based on the output of the camera and orientation sensor. These factors will improve the performance of positioning system greatly in accuracy, stability, and applicability.
(2) *Easier usage*. All required hardware is widely available in common smartphones. It is unnecessary to use deploy additional installations and users can use their smartphones for positioning in two modes: if high accuracy is unnecessary, they can choose Wi-Fi fingerprint positioning, otherwise, they can open the camera to improve the positioning results through the proposed sensors-calibrated algorithm.
(3) *Lower cost*. The proposed algorithm can be implemented on a smartphone. Firstly, we use the smartphone to locate, and on the other hand, we adopt crowd-sourcing via the smartphone to update the database. Equally important, we do not need to preset tags for image identification in the indoor scenario. However, if we do preset tags for image identification, it will be better for our algorithm.

For indoor positioning systems, it is ideal to achieve the optimal performance with the simplest equipment or method; however, there are many issues to be solved. Our proposed algorithm still has some shortcomings; fortunately, those shortcomings will be addressed in our future work:

Firstly, we should consider combining other built-in sensors in the smartphone. We have performed experiments under the assumption that the light is stable in indoor environments. In practice, the light might change as the weather changes. Smartphone cameras are sensitive to lighting and susceptible to this lowering the quality of the images. We will handle this aspect in the future by combining other sensors or other methods such as MoVIPS [26,27].

Secondly, the influence of equipment diversity should be considered. It is well known that the same built-in sensors in different smartphones have different performances, such as accuracy and stability, which will influence the positioning performance. We have tested the output value of the orientation sensor on the Huawei Honor 6 mobile phone and Motorola Moto X Pro mobile phone. The stability of the sensors in these two devices is obviously different. For simplification, we have done all experiments with the same brand of smartphone. Exploring the influence of equipment diversity for positioning will be a part of our future work.

Finally, the crowd-sourcing selection mechanism should also be considered. Crowd-sourcing is a suitable method to build a database for positioning. On the one hand, it can reduce the time needed to build the database. On the other hand, it can improve positioning performance as demonstrated by our experiments. However, the thing we should take into account is how can we filter out the unreliable data from all the uploaded data. At the same time, we will explore the quality control and management of crowd-sourcing to select better and more useful data to realize localization.

## Figures and Tables

**Table 1:** Storage format of the database.

Coordinate	AP Number	AP_1_	AP_2_	...	AP_m_	Image	Orientation Sensor Data
(x_1_,y_1_)	m_1_	MAC_m1_ = RSSI_m1_	MAC_m1_ = RSSI_m1_	...	MAC_m1_ = RSSI_m1_	1	{θ*_x_*_1_,θ*_y_*_1_,θ*_z_*_1_}
(x_2_,y_2_)	m_2_	MAC_m2_ = RSSI_m2_	MAC_m2_ = RSSI_m2_	...	MAC_m2_ = RSSI_m2_	2	{θ*_x_*_2_,θ*_y_*_2_,θ*_z_*_2_}
(x_3_,y_3_)	m_3_	MAC_m3_ = RSSI_m3_	MAC_m3_ = RSSI_m3_	...	MAC_m3_ = RSSI_m3_	3	{θ*_x_*_3_,θ*_y_*_3_,θ*_z_*_3_}
⋮	⋮	⋮	⋮	⋮	⋮	⋮	⋮
(x_n_,y_n_)	m_n_	MAC_mn_ = RSSI_mn_	MAC_mn_ = RSSI_mn_	...	MAC_mn_ = RSSI_mn_	n	{θ*_x_*_n_,θ*_y_*_n_,θ*_z_*_n_}

**Table 2:** Parameters for the 3D orientation sensor in the Huawei Honor 6.

Name	Supplier	Resolution	Heading		Pitch		Roll	
			Range	Accuracy	Range	Accuracy	Range	Accuracy
Inemo	STmicroelectronics	0.01°	0~360°	0.1°	−90°~+90°	0.1°	−180°~+180°	0.1°

**Table 3:** Parameters for the optical camera in the Huawei Honor 6.

Type	Orientation	Focal Length	View Angle	Size
BSI CMOS	90°	3.79 mm	63°	1920 × 1080

**Table 4:** Feature number and matching factors.

index	*F*_(*DB,i*)_	*F_c_*	η*_i_*	ζ*_i_*
(a) (b) (c)	659 643 628	647 563 489	1.888 1.664 1.464	0.853 0.128 0.018

**Table 5:** Time-consuming comparison of different algorithms.

Method	1	2	3	4	5	6	7	8
KWNN-based/ms	18.33	16.14	22.57	25.44	28.16	27.29	28.19	24.18
SURF + Wi-Fi/ms	1834.23	1898.58	1833.12	1777.30	1747.72	1716.91	1747.72	1908.65
Proposed/ms	1739.45	1734.18	1731.09	1699.88	1702.02	1691.47	1688.45	1743.09
